# Appendiceal abscess mimicking a painless abdominal wall mass in an adolescent: a case report

**DOI:** 10.3389/fmed.2026.1863642

**Published:** 2026-06-25

**Authors:** Guanghua Zhang, Ming Sun, Peng Huang, Hongqiang Bian, Jun Yang

**Affiliations:** 1 Department of General Surgery, Wuhan Children's Hospital, Tongji Medical College, Huazhong University of Science & Technology, Wuhan, China; 2 Department of Ophthalmology, Wuhan Children's Hospital, Tongji Medical College, Huazhong University of Science & Technology, Wuhan, China

**Keywords:** abdominal wall mass, appendicitis, atypical presentation, diagnostic error, diagnostic reasoning, pediatric surgery

## Abstract

**Background:**

Acute appendicitis is the most common surgical abdominal emergency in children. Its atypical presentations remain a significant source of diagnostic error. The presentation of an appendiceal abscess as an isolated abdominal wall mass, in the complete absence of abdominal pain, represents a misleading manifestation that directly contradicts the classic teaching that peritoneal signs are essential for diagnosis.

**Case presentation:**

A previously healthy 13-year-old boy presented with a 10-day history of a painful right lower abdominal wall swelling, explicitly denying any abdominal pain. Initially misdiagnosed with a soft tissue infection based on a focused abdominal wall ultrasound, he was referred after 10 days of unsuccessful treatment. A non-contrast computed tomography scan revealed a secondary abdominal wall abscess connected via an inflammatory tract to a large periappendiceal abscess. Management followed a staged strategy: intravenous antibiotics led to resolution, followed by an uncomplicated delayed laparoscopic appendectomy 3 months later. The patient recovered fully.

**Interpretation:**

This case illustrates a critical diagnostic pitfall where the absence of expected abdominal pain and a prominent local finding led to initial misdiagnosis as soft-tissue infection. This case highlights the diagnostic challenge posed by this presentation and serves to remind clinicians of the importance of considering an intra-abdominal source and obtaining a comprehensive ultrasound evaluation in children with an unexplained abdominal wall mass, even in the absence of abdominal pain. A diagnostic consideration pathway is presented as a teaching schematic to illustrate a systematic approach to evaluation in such ambiguous scenarios. It is crucial to emphasize that this pathway is derived from a single case and is presented for educational purposes, not as a validated clinical tool.

## Highlights

- An isolated, painful abdominal wall mass in a child with marked systemic inflammation should always prompt consideration of an occult intra-abdominal source, even in the complete absence of abdominal pain.
- Protocol adherence is critical: a comprehensive abdominal and pelvic ultrasound, rather than a focused examination of the symptomatic area, is essential to reduce the risk of overlooking occult intra-abdominal pathology.
- In delayed, high-risk presentations with failed initial management, cross-sectional imaging (CT or MRI) is justified as a second-line step after comprehensive abdominal ultrasound to definitively exclude complex intra-abdominal pathology when ultrasound is non-diagnostic or discordant with clinical findings.
- Staged management—initial source control with antibiotics followed by interval appendectomy—is safe and effective for complex appendicitis, even when diagnosis has been significantly delayed.
- In atypical presentations, adherence to established imaging guidelines (e.g., comprehensive ultrasound first) and maintaining a broad differential diagnosis are essential to avoid diagnostic delay.

## 1 Introduction

Acute appendicitis dominates pediatric surgical emergencies, yet its atypical presentations remain a persistent source of diagnostic error (1, 2). Among these, an isolated abdominal wall mass without abdominal pain is particularly treacherous, as it contradicts the teaching that peritoneal irritation is essential for diagnosis. While contiguous spread from perforated appendicitis to the abdominal wall is documented (3–5), presentations completely lacking abdominal pain are rare and pose a challenge to clinical recognition.

We present this instructive case of a “silent” appendiceal abscess manifesting solely as an abdominal wall mass. We use it to examine the diagnostic assumptions that contributed to initial misdiagnosis and to illustrate a structured reasoning process, formalized as a diagnostic consideration pathway, intended to enhance diagnostic vigilance for similar presentations.

## 2 Case presentation

Core clinical summary:

- Patient: 13-year-old, previously healthy boy
- Chief Complaint: Painful right lower abdominal wall swelling for 10 days. Explicit denial of abdominal pain
- Key Physical Finding: Tender, erythematous 3 × 5 cm abdominal wall mass. Otherwise soft, non-tender abdomen
- Salient Lab Finding: Marked leukocytosis (22.50 × 10^9^/L) and elevated inflammatory markers (CRP 31 mg/L, PCT 0.46 ng/mL)
- Diagnostic Turning Point: Non-contrast CT revealed an abdominal wall abscess connected via a tract to a large periappendiceal abscess
- Management: Staged approach: IV antibiotics → resolution → interval laparoscopic appendectomy (3 months later)
- Outcome: Full recovery, no recurrence at 6 months.

### 2.1 History and presentation

A previously healthy 13-year-old boy (35 kg) presented to our emergency department in 2025 with a 10-day history of a progressively painful, localized swelling in his right lower abdomen. He was adamant that he had no abdominal pain, cramping, nausea, or fever. There was no history of trauma. He had been evaluated and managed at an outside facility where a diagnosis of soft-tissue infection was made, but the 10-day course of treatment there provided no relief.

### 2.2 Physical examination

Vital signs were stable. Examination revealed a 3 × 5 cm, erythematous, firm, and exquisitely tender mass in the right lower abdominal wall. Crucially, deep palpation away from the mass revealed no tenderness, no guarding, no rebound tenderness, and normal bowel sounds. The abdominal examination was, aside from the visible and palpable mass, entirely benign.

### 2.3 Laboratory investigations

Markers of systemic inflammation were strikingly elevated: leukocyte count 22.50 × 10^9^/L (neutrophils 79.5%), high-sensitivity C-reactive protein 31 mg/L, and procalcitonin 0.46 ng/mL. Routine biochemistry was normal. Serum lactate was not measured, as the primary clinical concern was localized infection rather than bowel ischemia or septic shock.

### 2.4 Imaging studies and diagnostic turning point

The referral report indicated an abdominal wall ultrasound focused solely on the subcutaneous collection but did not note any intra-abdominal pathology. The profound discrepancy between significant systemic inflammation and a seemingly localized finding, coupled with the failure of prior treatment, prompted immediate and comprehensive investigation. Given the high clinical suspicion for a complex, occult intra-abdominal source in this delayed and systemically ill presentation, a non-contrast abdominal CT scan was performed. This modality was selected to rapidly and comprehensively evaluate for an abscess and its possible tract morphology while avoiding contrast-associated risks in a pediatric patient. We acknowledge that contrast-enhanced CT or MRI might have provided additional detail on vascular involvement and abscess septation. The non-contrast study was selected to minimize procedural complexity and contrast-associated risks in this pediatric patient, and it provided sufficient diagnostic clarity (revealing the abscess, tract, and gas) to guide immediate management. The CT scan provided definitive clarity (Figures 1A–D), revealing: (1) a right lower abdominal wall abscess (approx. 30 × 50 × 50 mm); (2) a large periappendiceal phlegmon/abscess (approx. 76 × 62 × 155 mm); and (3) a clear inflammatory tract connecting the two, confirming the appendiceal origin.

**Figure 1:**
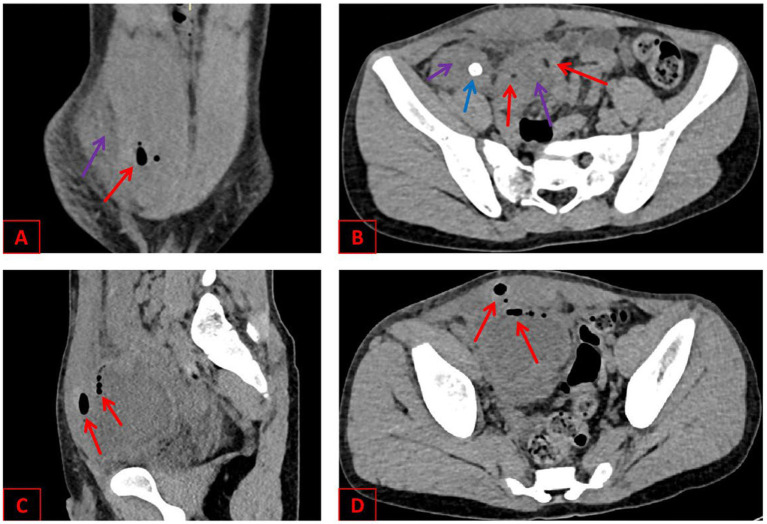
Serial axial and coronal non-contrast CT images of the abdomen. **(A)** Abdominal wall abscess with intramuscular (purple arrow) and subcutaneous involvement and intralesional gas (red arrow). **(B)** Periappendiceal abscess (purple arrow) with gas (red arrow), and adjacent thickened appendix with appendicolith (blue arrow). **(C, D)** Key Signs: Gas-containing inflammatory tract (red arrows) directly connects the abdominal wall and periappendiceal abscesses, confirming appendiceal origin.

### 2.5 Final diagnosis and differential diagnosis

#### 2.5.1 Final diagnosis

- Secondary abdominal wall abscess.
- Acute appendicitis with perforation and periappendiceal abscess.

#### 2.5.2 Differential diagnosis

Prior to definitive CT diagnosis, the differential diagnosis for this isolated abdominal wall mass with marked systemic inflammation included: (1) primary soft-tissue infection/abscess (the initial misdiagnosis); (2) abdominal wall hematoma (trauma was denied); (3) incarcerated Spigelian or other abdominal wall hernia with strangulation; (4) soft-tissue neoplasm with secondary infection; and (5) an occult intra-abdominal process with fistulous extension to the abdominal wall. The combination of a significant systemic inflammatory response in the setting of a localized finding, along with the lack of clinical improvement despite prior therapy, raised concern for a more complex source. This suspicion, coupled with the absence of trauma history, shifted the differential diagnosis toward an occult intra-abdominal process, prompting the decision to proceed with cross-sectional imaging.

### 2.6 Management and outcome

#### 2.6.1 Initial phase: infection source control

The patient was started on intravenous meropenem (20 mg/kg per dose, every 8 h), chosen for its broad-spectrum coverage against likely polymicrobial flora in a healthcare-associated, complicated intra-abdominal infection. His pain and swelling improved markedly within 72 h. Inflammatory markers normalized over the following week, and the wall abscess resolved completely without drainage. No changes to the initial therapeutic plan were required during treatment. The entire staged management strategy was implemented as planned due to the satisfactory resolution of inflammation and abscess with intravenous antibiotic therapy.

#### 2.6.2 Second phase: definitive surgery

In alignment with WSES guidelines for complex appendicitis (6), an interval laparoscopic appendectomy was performed 3 months later. The decision for delayed surgery was based on the resolution of acute inflammation and the presence of a well-contained abscess, to avoid the higher morbidity associated with immediate surgery in a septic field. The procedure was straightforward. Intraoperatively, only mild adhesions around the appendix and cecum were identified and gently separated. No inflammatory fistula was observed during the laparoscopic exploration. The appendix presented obvious fibrosis and thickening (Figure 2). Histopathological examination confirmed chronic transmural appendicitis.

**Figure 2:**
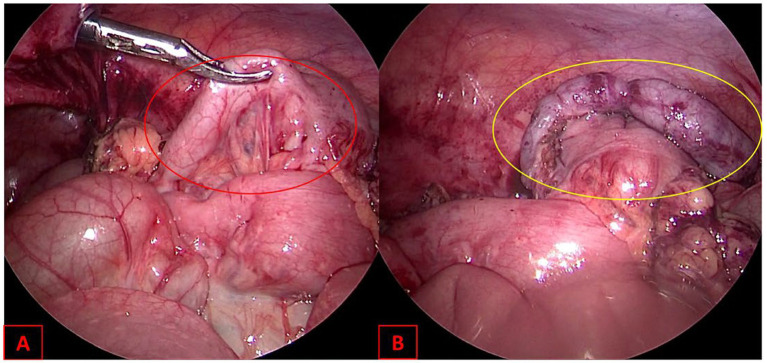
Serial intraoperative laparoscopic images. **(A)** Mild adhesions surrounding the appendix and cecum; **(B)** The fibrotic and thickened appendix after separation. No inflammatory fistula was detected throughout the operation.

#### 2.6.3 Follow-up and patient perspective

The patient remained asymptomatic with no evidence of recurrence at six-month follow-up. Follow-up imaging surveillance was appropriately performed using ultrasound.

Patient perspective:

The patient and family provided verbal feedback expressing satisfaction with the staged management approach and full recovery. They reported appreciation for the comprehensive evaluation that led to correct diagnosis after initial misdiagnosis, and confirmed adherence to follow-up recommendations.

### 2.7 Timeline of clinical course

A consolidated timeline of the diagnosis, management, and follow-up is presented in Table 1.

**Table 1:** Timeline of the clinical course, diagnosis, and management.

Time point	Key event	Clinical/management action	Outcome/status
Day 1–10	Symptom onset and initial care	Painful right lower abdominal wall mass developed. Focused abdominal wall ultrasound performed; diagnosed as soft tissue infection at an outside hospital.	Treatment failed, symptoms persisted.
Day 10	Transfer to our hospital	Emergency referral due to treatment failure. Localized abdominal wall mass with marked leukocytosis (WBC 22.50 × 10^9^/L). No abdominal pain.	Systemic inflammatory response evident.
Day 10	Definitive diagnosis	Comprehensive non-contrast abdominal CT scan performed.	Diagnosis confirmed: secondary abdominal wall abscess connected to a large periappendiceal abscess.
Day 10–17	Initial infection control	Intravenous meropenem initiated.	Pain and swelling markedly improved within 72 h. Inflammatory markers normalized within 1 week.
3 months later	Definitive surgery	Interval laparoscopic appendectomy performed.	Uneventful surgery. Histopathology confirmed chronic appendicitis.
Post-op month 6	Follow-up	Clinical and ultrasonographic surveillance.	Asymptomatic, no recurrence, full recovery.

## 3 Discussion

### 3.1 The diagnostic pitfall of “required” pain

This case illustrates an uncommon presentation of appendiceal abscess in which abdominal pain was entirely absent: the expectation that significant appendiceal pathology is invariably painful within the abdomen. The absence of abdominal pain contributed to the initial attribution of the lesion to a localized abdominal wall process, delaying consideration of an intra-abdominal source (7, 8).

Compared to previously reported cases of appendiceal abscess presenting as an abdominal wall mass (3–5), our case is distinctive in two respects: first, the complete absence of abdominal pain throughout the 10-day course, whereas prior cases typically reported some degree of abdominal discomfort; second, the initial diagnostic error was driven by a focused rather than comprehensive ultrasound examination, highlighting that this case also suggests that broader imaging evaluation may be helpful in atypical presentations with discordant clinical findings.

### 3.2 Focused ultrasound in atypical presentations

The initial ultrasound evaluation was appropriately focused on the presenting complaint but did not include a systematic survey of the deeper abdomen. In a typical soft-tissue infection, this focused approach would be sufficient; however, in this atypical presentation (isolated wall mass with marked systemic inflammation and no trauma), a broader anatomic survey was warranted. The critical lesson is protocol selection: ACR Appropriateness Criteria® (9) and the 2024 IDSA guidelines (10) recommend comprehensive abdominal and pelvic ultrasound as the initial imaging for children with suspected intra-abdominal infection; application of this principle to our patient’s atypical presentation might have detected the intra-abdominal pathology and prevented the delay. Instead, the focused examination, while adequate for its intended scope, could not evaluate for the occult intra-abdominal source that the clinical picture suggested.

In this case, the non-contrast CT served as a definitive diagnostic study (11), providing a broad anatomic view that systematically identified the intra-abdominal pathology that the initial assessment, constrained by its focused scope, could not address.

### 3.3 Ultrasound limitations and protocol selection

Point-of-care ultrasound (POCUS) is valuable for appendicitis screening (12), but its accuracy is operator-dependent and limited in complex cases (12, 13). When evaluation is focused primarily on a superficial process, a focused ultrasound may fail to survey the deeper abdomen. This highlights the importance of selecting the appropriate examination protocol based on clinical presentation: a focused protocol suffices for typical presentations, but atypical scenarios with discordant systemic inflammation require comprehensive evaluation. In cases with marked systemic inflammation and persistent diagnostic uncertainty, broader evaluation may be warranted despite a limited focused ultrasound finding. Our intent is not to critique individual practice but to illustrate how selecting a protocol for comprehensive anatomic evaluation can prevent such diagnostic pitfalls in appropriately identified high-risk scenarios.

### 3.4 Pathophysiology of a silent perforation

The pathophysiology likely involves a contained, retrocecal perforation with inflammation tracking preferentially along fascial planes to the abdominal wall, rather than causing diffuse peritonitis (3–5, 14). This containment explains the absence of peritoneal signs, while inflammation of somatic nerves in the abdominal wall accounted for the localized pain. The retrocecal location, present in up to 25% of appendicitis cases, predisposes to atypical presentations and delayed diagnosis due to shielding from anterior peritoneal irritation (14).

### 3.5 Application of a staged management approach in an atypical presentation

The successful outcome demonstrates the applicability of the staged management strategy described in current guidelines (6, 15) to this highly atypical presentation. This approach prioritizes avoiding the high morbidity of emergency surgery in a septic field, and this case reinforces that adherence to established protocols remains effective even when diagnosis has been significantly delayed.

### 3.6 A diagnostic consideration pathway for teaching

Building upon the lessons of this case, we synthesized the clinical reasoning into a diagnostic consideration pathway (Figure 3). It is crucial to emphasize that this pathway is derived from and illustrative of this single case. It is presented not as a validated clinical tool, but as a visual schematic to make explicit the stepwise application of established principles—primarily the ALARA mandate for ultrasound-first imaging—to a high-risk, atypical scenario.

**Figure 3:**
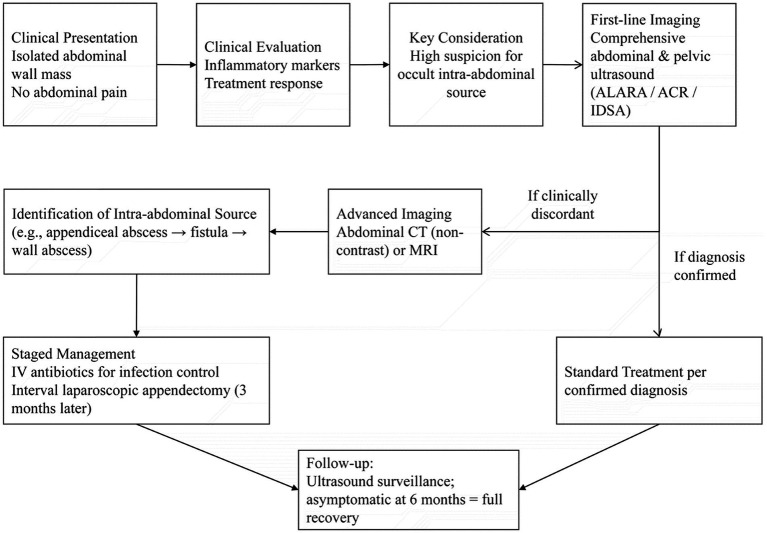
Diagnostic consideration pathway for a child presenting with an isolated abdominal wall mass and no abdominal pain. This pathway synthesizes the clinical reasoning applied in the present case, illustrating the application of established imaging principles (e.g., ALARA, ACR guidelines, IDSA 2024 recommendations) to this specific high-risk scenario. It emphasizes a high index of suspicion for an intra-abdominal source. The first imaging step is a comprehensive abdominal and pelvic ultrasound. Advanced cross-sectional imaging (CT or MRI) is considered when ultrasound is non-diagnostic and clinical concern remains high due to significant systemic inflammation or lack of improvement, to evaluate for occult intra-abdominal pathology. This diagnostic consideration pathway is a teaching schematic derived from a single case. It is not a validated clinical tool and is not intended for direct clinical decision-making.

The pathway begins with consideration of a possible intra-abdominal source for an intra-abdominal source for any non-traumatic abdominal wall abscess. The first imaging step is a comprehensive abdominal and pelvic ultrasound, consistent with the ultrasound-first approach recommended by ACR Appropriateness Criteria® (9) and the 2024 IDSA guidelines (10) for children with suspected intra-abdominal infection, and in keeping with the ALARA mandate (16). When ultrasound is non-diagnostic but clinical concern remains high due to significant systemic inflammation or failure to improve, consideration of advanced cross-sectional imaging (CT or MRI) is warranted to exclude a complex intra-abdominal process.

### 3.7 Rationale for advanced imaging in delayed, high-risk presentations

Current guidelines recommend ultrasound as the initial imaging modality for children with suspected intra-abdominal infection, reserving CT or MRI for when ultrasound is inconclusive or clinical concern persists (9, 10, 16). This case underscores that principle: while a focused abdominal wall ultrasound was initially performed, a comprehensive abdominal and pelvic survey—as per protocol—might have identified the occult source earlier.

The patient’s presentation, however, was marked by a 10-day delay, failed prior treatment, and significant systemic inflammation, creating a high-risk scenario demanding urgent diagnostic certainty. Consequently, non-contrast CT was employed as a second-line, cross-sectional tool to rapidly exclude a complex intra-abdominal source and guide management. This decision balanced the imperative to minimize radiation exposure against the immediate need for definitive risk stratification in a systemically unwell child.

We acknowledge that intravenous contrast could have provided additional anatomical detail regarding abscess wall enhancement (10). The non-contrast CT, however, was sufficient to establish the diagnosis and delineate the inflammatory tract, and we transparently report the non-use of contrast as a limitation. All follow-up surveillance was performed with ultrasound.

### 3.8 Strengths and limitations

The primary strength of this report is the documentation of an instructive presentation of a common disease, supported by complete imaging documentation and clinical follow-up. The synthesis of the clinical reasoning into a diagnostic consideration pathway provides a clear educational takeaway, explicitly framed as a derivative teaching tool.

The limitations are those inherent to a single case report. The pathway is a conceptual schematic derived from this single experience and lacks prospective validation. The retrospective nature limits our ability to fully characterize the initial ultrasound examination—we were unable to review the original ultrasound images or assess the operator expertise and technical conditions at the outside facility, and our interpretation is therefore subject to hindsight bias. Additionally, while non-contrast CT was diagnostic, the use of intravenous contrast might have provided further anatomical detail regarding abscess enhancement.

## 4 Conclusion

This case report highlights a highly atypical and deceptive presentation of appendicitis, which manifested solely as an isolated abdominal wall mass without abdominal pain. The initial diagnosis of soft tissue infection was understandable given the prominent abdominal wall findings and absence of abdominal pain. However, the persistent systemic inflammatory response and treatment failure ultimately prompted broader evaluation. The key lessons are: (1) an unexplained abdominal wall mass with systemic inflammation mandates consideration of an intra-abdominal source; (2) initial imaging should include consideration of a comprehensive abdominal and pelvic ultrasound when systemic inflammation appears disproportionate to an apparently localized abdominal wall process, not a focused examination; and (3) in high-risk scenarios with diagnostic uncertainty, timely cross-sectional imaging is warranted. The accompanying diagnostic consideration pathway (Figure 3) visually summarizes this structured approach, serving as an educational tool to reinforce guideline-adherent evaluation in similar challenging presentations.

## Data Availability

The original contributions presented in the study are included in the article/supplementary material, further inquiries can be directed to the corresponding author/s.

## Author’s note

This case report was prepared in accordance with the CARE guidelines.
